# Supplementary material for: Blood-Epigenetic Biomarker Associations with Tumor Immunophenotype in Patients with Urothelial Carcinoma from JAVELIN Bladder 100
Source: Cancers (Basel). 2025 Jul 14;17(14):2332. doi: 10.3390/cancers17142332 (PMC12293378; doi:10.3390/cancers17142332)

Supplementary Table S1. Characteristics of the biomarker analysis set compared with the full analysis set. BSC, best supportive care; **CR**, complete response; **OBD**, Oxford BioDynamics; **PR**, partial response; **SD**, stable disease; **St. Dev.**, standard deviation.

	**Full analysis set**		**OBD analysis set**	
	**Avelumab plus BSC (N=355)**	**BSC (N=345)**	**Avelumab plus BSC (n=262)**	**BSC (n=234)**
**Age, years**				
Median (IQR)	69 (61-74)	69 (62-74)	69 (62-75)	69 (62-74)
Mean (St. Dev.)	67.3 (9.5)	67.7 (9.2)	67.8 (9.2)	67.8 (9.1)
**Race, n (%)**				
Asian	75 (21.1)	81 (23.5)	45 (17.2)	46 (19.7)
Black or African American	2 (0.6)	0	2 (0.8)	0
Other	21 (5.9)	15 (4.3)	20 (7.6)	14 (6)
Unknown	20 (5.6)	16 (4.6)	18 (6.9)	14 (6)
White	237 (66.8)	233 (67.5)	177 (67.6)	160 (68.4)
**Ethnicity, n (%)**				
Hispanic or Latino	18 (5.1)	12 (3.5)	7 (2.7)	5 (2.1)
Not Hispanic or Latino	291 (82)	293 (84.9)	214 (81.7)	196 (83.8)
Not reported	42 (11.8)	36 (10.4)	38 (14.5)	30 (12.8)
Unknown	4 (1.1)	4 (1.2)	3 (1.1)	3 (1.3)
**Sex, n (%)**				
Female	85 (23.9)	74 (21.4)	62 (23.7)	48 (20.5)
Male	270 (76.1)	271 (78.6)	200 (76.3)	186 (79.5)
**Best response to first-line chemotherapy and metastatic disease site at first-line chemotherapy, n (%)**				
CR or PR and nonvisceral	115 (32.4)	110 (31.9)	83 (31.7)	74 (31.6)
CR or PR and visceral	142 (40)	138 (40)	105 (40.1)	92 (39.3)
SD and nonvisceral	46 (13)	47 (13.6)	35 (13.4)	31 (13.2)
SD and visceral	52 (14.6)	50 (14.5)	39 (14.9)	37 (15.8)

Supplementary Table S2. List of 15 markers passing the JAV-Immuno, TMB, and/or treatment interaction criteria. Twenty-five peripheral blood markers that met criteria for predicting JAV-Immuno levels in tumor were further screened for potential interactions with TMB and treatment based on OS outcome. The effect column indicates whether a marker/dilution pair was selected based on Cox models for its main effect (1-Main), its interaction with treatment alone (2-Way), or its interaction with treatment and TMB (3-way). The p values were based on 2-sided Wald test. (See the Methods section for details of the pre-filtering and modeling steps). Some chromosomal loci were covered by >1 marker from the original 150-marker set as indicated. Genes in bold font were more strongly expressed in the lymphoid aggregate region relative to the epithelial region of the bladder cancer specimen shown in Figure 3d. **OS**, overall survival; **TMB**, tumor mutation burden.

Chromosome	Start	End	Effect	Marker	Dilution	p value	Genes included in locus	No. of markers covering locus in original marker set
9	108756405	108869639	1-Main	OBD175265267	12	0.064	***ACTL7A***, *ACTL7B*, *IKBKAP*	3
22	20718358	20985871	2-Way	OBD175005007	12	0.028	*AIFM3*, *CRKL*, ***LZTR1***, *PI4KA*, ***SERPIND****1*, *SNAP29*	4
8	88020609	88141099	2-Way	OBD175025027	14	0.071	***CNBD1***, *DCAF4L2*, *MMP16*	1
16	35636206	35720319	2-Way	OBD175037039	12	0.005	*RP11-812E19.9*, *TP53TG3B*, *TP53TG3C*	5
22	20718358	20859143	2-Way	OBD175061063	12	0.063	*PI4KA*, ***SERPIND1***, *SNAP29*	4
15	62189008	62247072	2-Way	OBD175161163	14	0.022	***C2CD4A***, ***C2CD4B***, *VPS13C*	4
3	152119516	152223492	3-Way	OBD175133135	1	0.098	***MBNL1***, *SUCNR1*, *TMEM14E*	1
10	46488771	46553191	3-Way	OBD175217219	1	0.092	*ANXA8L1*, *GPRIN2*, *NPY4R*	3
19	42130144	42190651	3-Way	OBD175269271	14	0.016	*DEDD2*, ***POU2F2***, *ZNF526*	1
15	82455628	82552450	3-way	OBD175373375	12	0.02	***CPEB1***, *RP11-152F13.10*, *RPS17*	1
22	20718358	20982169	3-Way	OBD175393395	12	0.009	*AIFM3*, *CRKL*, ***LZTR1***, *PI4KA*, ***SERPIND1***, *SNAP29*	4
2	6903602	7063423	3-Way	OBD175509511	12	0.091	*CMPK2*, *RNF144A*, *RSAD2*	5
16	58903156	59007164	3-Way	OBD175645647	1	0.043	*CNOT1*, *GOT2*, *SLC38A7*	6
3	100637059	100696938	3-Way	OBD175697699	14	0.049	***ABI3BP***, *GPR128*, *TFG*	1
19	37677698	37739114	3-Way	OBD175729731	12	0.089	*CTD-2528L19.4*, *ZFP30*, *ZNF573*, *ZNF607*, *ZNF781*	3

Supplementary Figure S1. Characterization of CCMs emerging from screening and modeling workflows. A. CONSORT flow diagram of patient disposition. Biomarker analyses were performed on specimens from patients who both (1) received ≥1 dose of study drug in the avelumab + BSC arm or completed cycle 1 day 1 in the BSC arm (ie, the safety set) and (2) had tumor transcriptome and chromatin marker data available. **B.** Prevalence and linkage of markers passing pre-filtering criteria for multivariate modeling. i. For each marker, prevalence was calculated as the proportion of patients with the marker present among all patients analyzed (N=496). ii. Phi coefficient (measure of association between binary variables) matrix among pre-filtered markers. **C.** Map of genes covered by chromatin markers. Circles represent genes covered by at least 1 marker. Chromosome and starting positions of marker loci or individual genes (from JAV-Immuno) were assigned using hg38 coordinates. Selected: markers passing TMB modeling criteria. Not selected: remaining markers from original array screen. **BSC**, best supportive care; **CCM**, chromatin conformation marker; **Chr**, chromosome; **TMB**, tumor mutation burden.

A

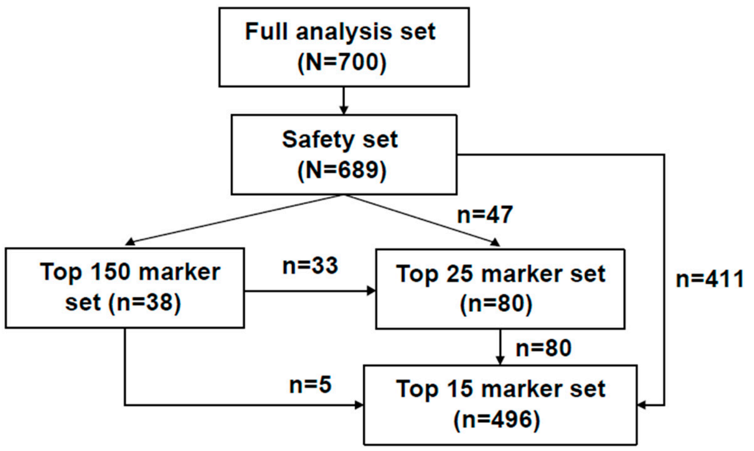

B

i.

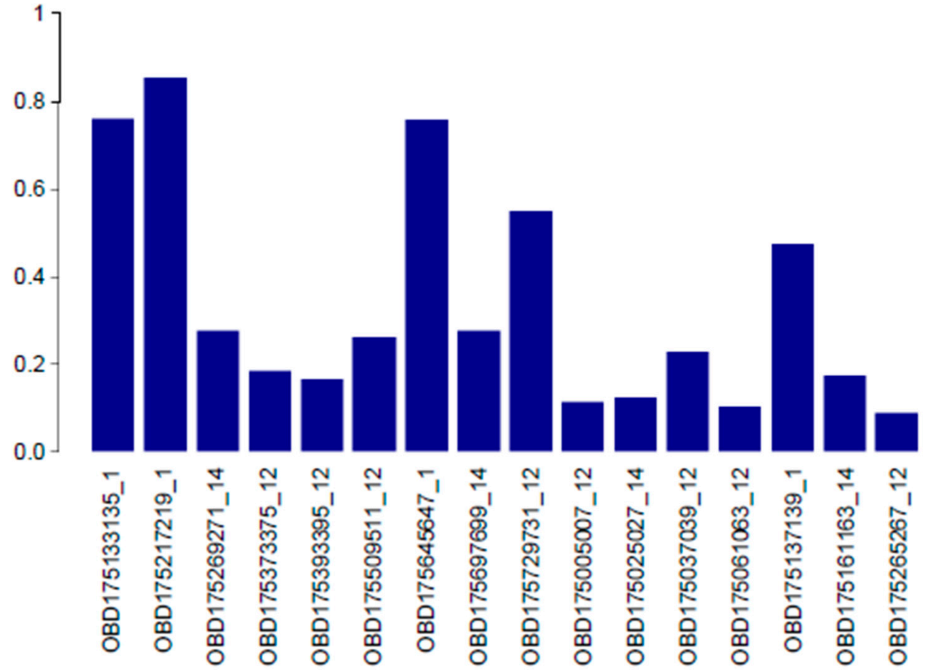

1
0.8
0.6
0.4
0.2
0.0
OBD175133135_1
OBD175217219_1
OBD175269271_14
OBD175373375_12
OBD175393395_12
OBD175509511_12
OBD175645647_1
OBD175697699_14
OBD175729731_12
OBD175005007_12
OBD175025027_14
OBD175037039_12
OBD175061063_12
OBD175137139_1
OBD175161163_14
OBD175265267_12

ii.

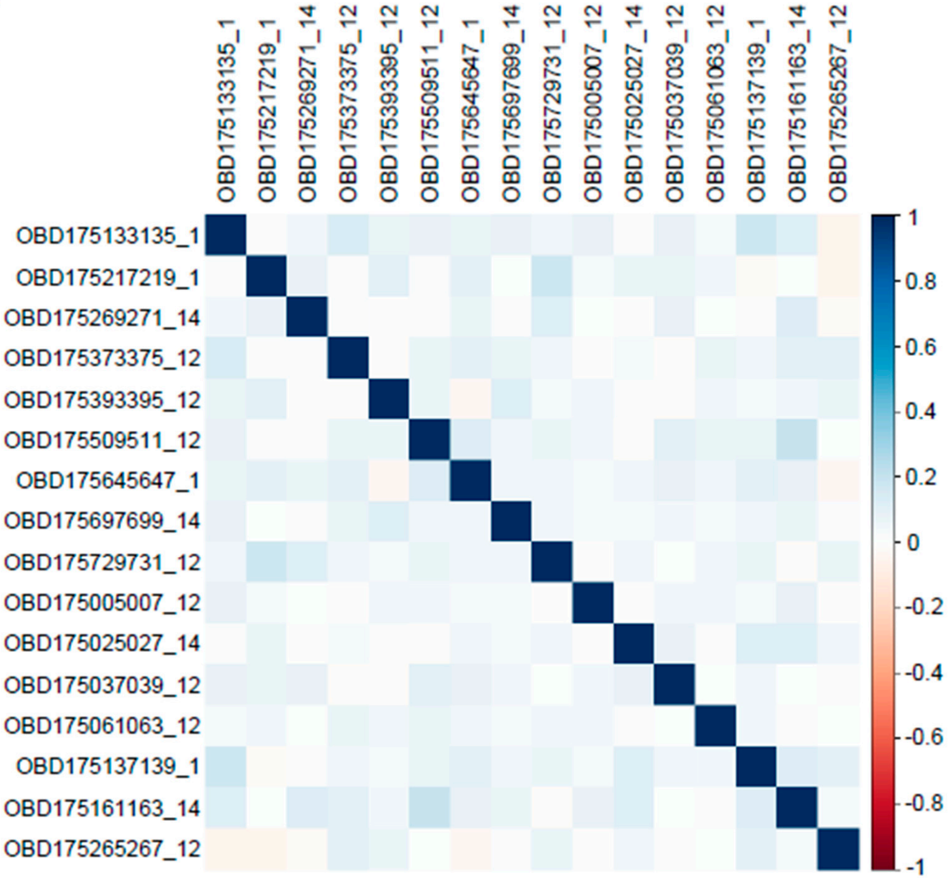

OBD175133135_1
OBD175217219_1
OBD175269271_14
OBD175373375_12
OBD175393395_12
OBD175509511_12
OBD175645647_1
OBD175697699_14
OBD175729731_12
OBD175005007_12
OBD175025027_14
OBD175037039_12
OBD175061063_12
OBD175137139_1
OBD175161163_14
OBD175265267_12
1
0.8
0.6
0.4
0.2
0
-0.2
-0.4
-0.6
-0.8
-1

C

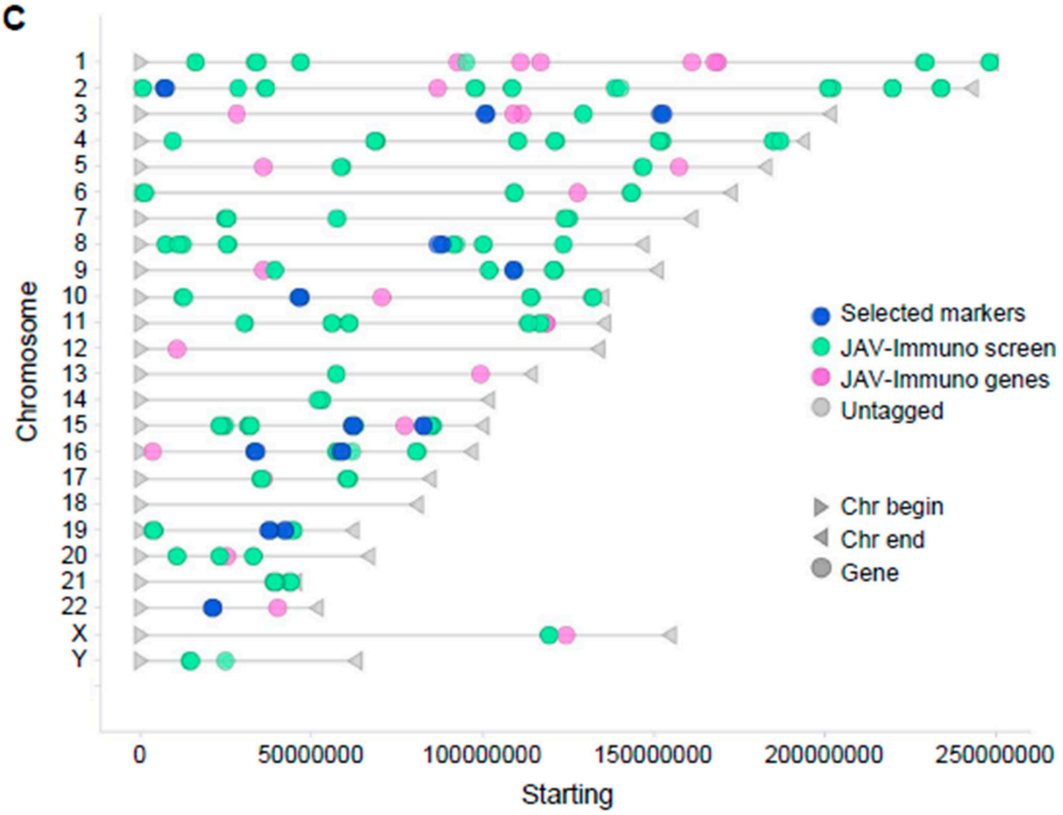

	Selected	Not selected
Number of genes covered by all markers in the set	44	323
Number of genes covered by 1 marker	15	228
Number of genes covered by >1 marker (% of genes covered)	29 (66)	95 (29)

Supplementary Figure S2. OS and PFS outcomes with the full analysis set and biomarker analysis set. **OS**, overall survival; **PFS**, progression-free survival.

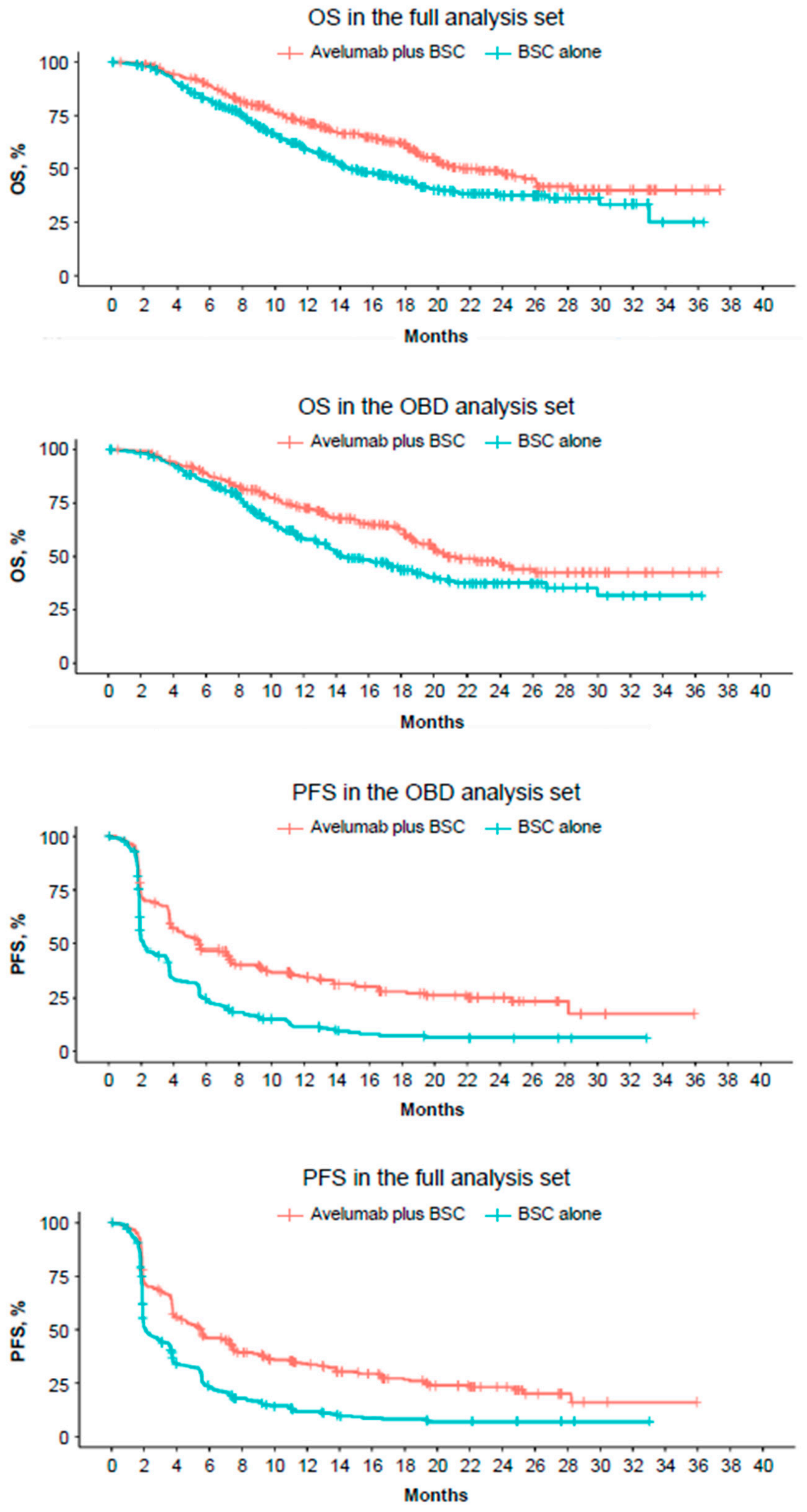

Supplementary Figure S3. Identification of optimal hyperparameter pairs for elastic modeling to select markers interacting with TMB and/or treatment on OS outcome. For each round of 5-fold cross-validation, an optimal pair of α and λ associated with minimal model error was identified. The same procedure was iterated 1000 times to account for potential cross-validation error. The frequency distribution of the optimal hyperparameter pairs was plotted in a Hexbin plot with color indicating the number of optimal hyperparameter pairs located inside each hexagon. The most frequently selected hyperparameter pairs from the hexagon with the largest count (red arrow) was chosen as optimal and was applied to the full dataset to obtain the final models.
BSC, best supportive care; **OS**, overall survival; **PFS**, progression-free survival, **OBD**, Oxford BioDynamics; **TMB**, tumor mutation burden.

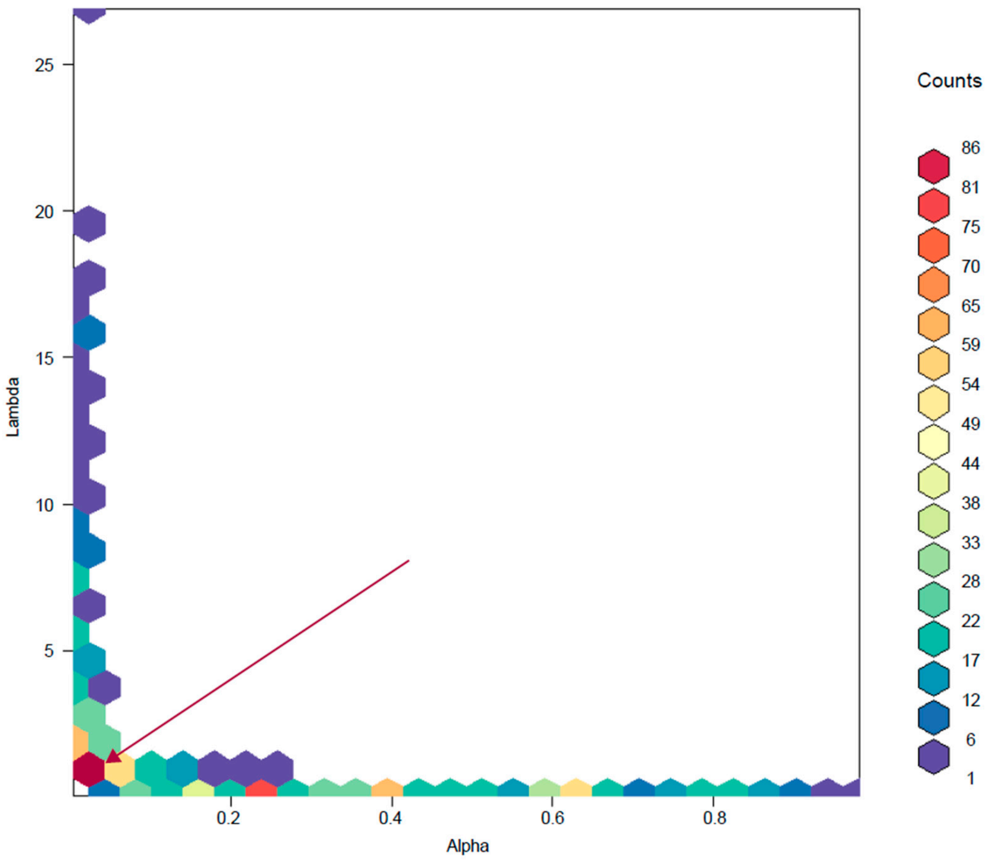

Supplementary Figure S4. Heterogeneous expression of genes in a single marker across multiple cell types in Tabula Sapiens. A. immune. **B**. Endothelial. **C**. stromal. **D**. Epithelial. Cell types from the CURIE knowledge graph were mapped to the cell types reported [24]. The color represents aggregation by mean normalized by z-score. The size of circles is proportional to the percentage of cells with non-zero expression. **NK**, natural killer.

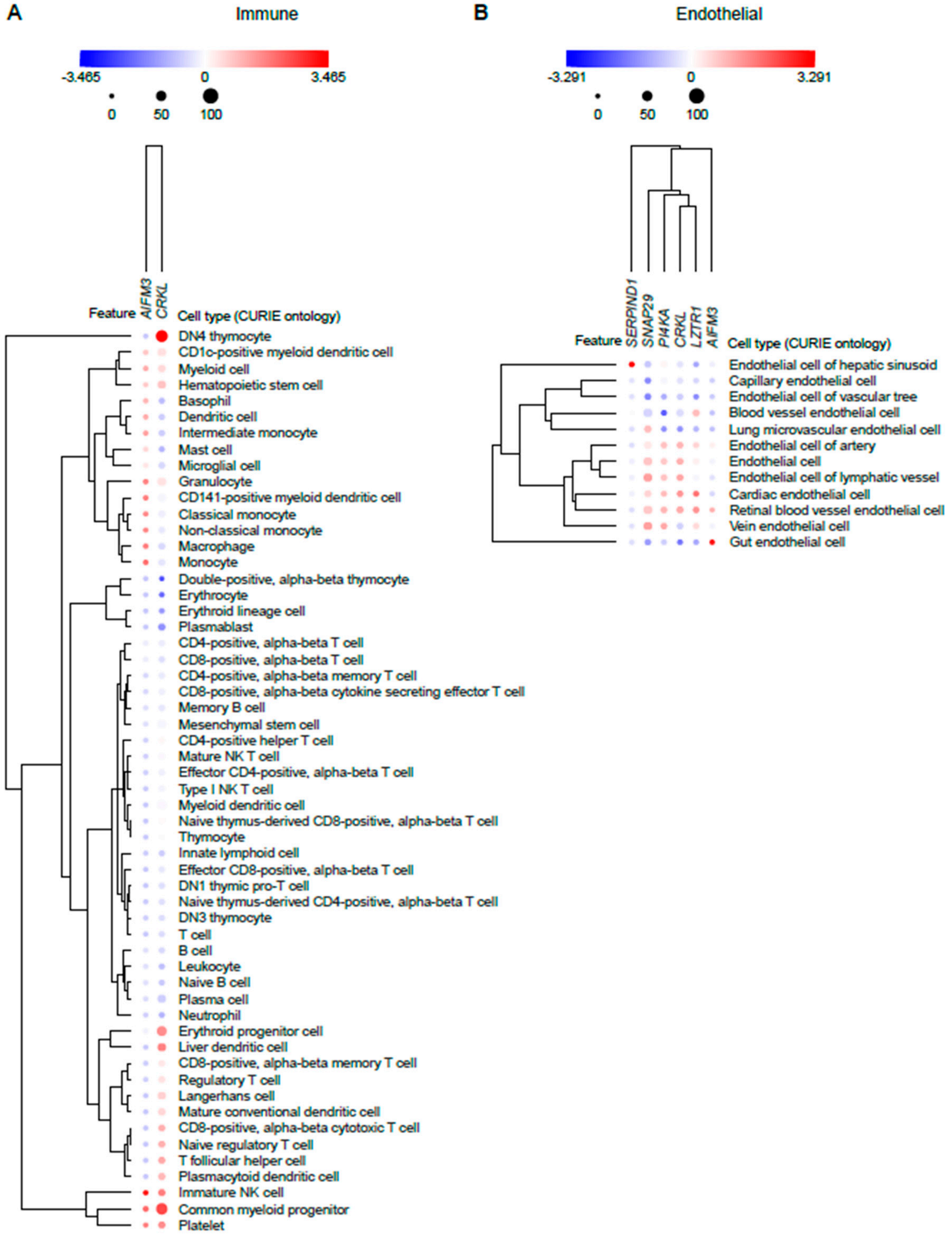

C

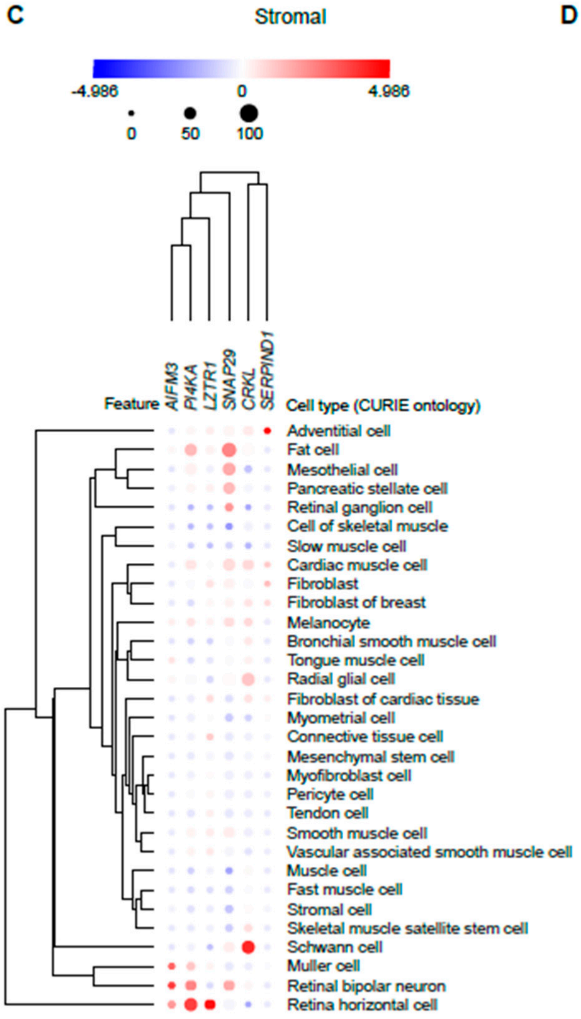
Stromal
-4.986
0
4.986
0
50
100
Feature
AIFM3
PI4KA
LZTR1
SNAP29
CRKL
SERPIND1
Cell type (CURIE ontology)
Adventitial cell
Fat cell
Mesothelial cell
Pancreatic stellate cell
Retinal ganglion cell
Cell of skeletal muscle
Slow muscle cell
Cardiac muscle cell
Fibroblast
Fibroblast of breast
Melanocyte
Bronchial smooth muscle cell
Tongue muscle cell
Radial glial cell
Fibroblast of cardiac tissue
Myometrial cell
Connective tissue cell
Mesenchymal stem cell
Myofibroblast cell
Pericyte cell
Tendon cell
Smooth muscle cell
Vascular associated smooth muscle cell
Muscle cell
Fast muscle cell
Stromal cell
Skeletal muscle satellite stem cell
Schwann cell
Muller cell
Retinal bipolar neuron
Retina horizontal cell

D

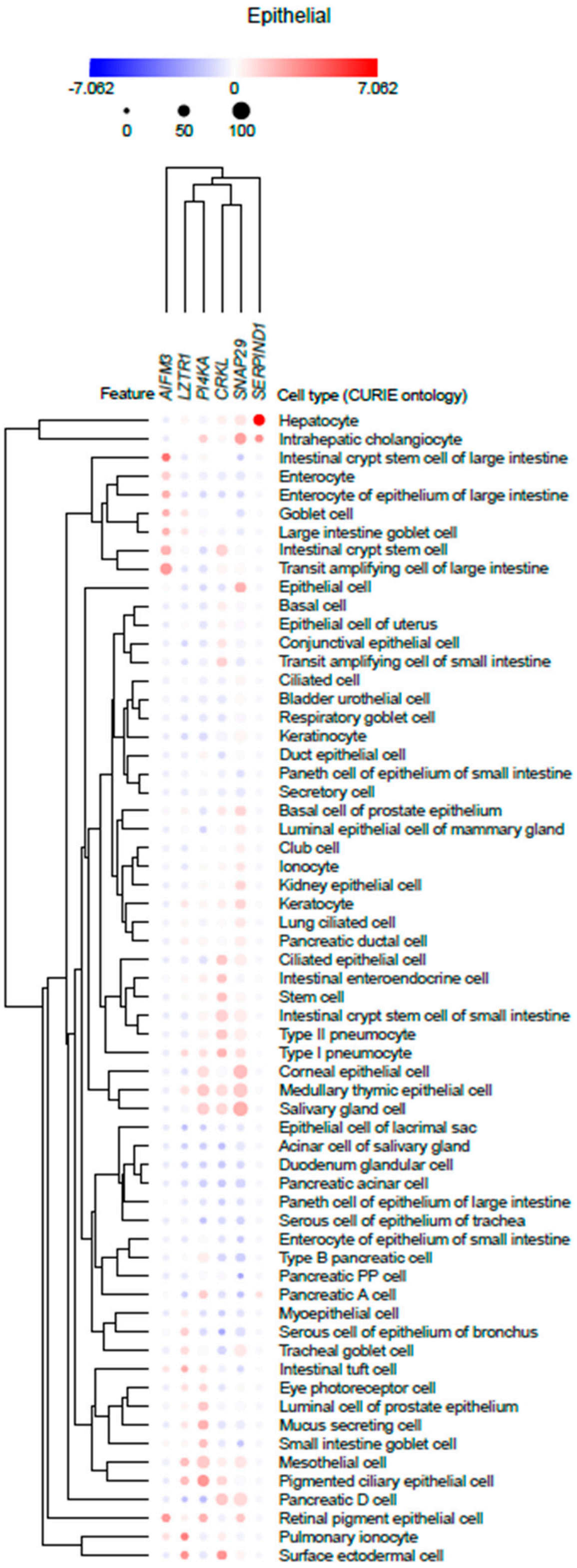
Epithelial
-7.062
0
7.062
0
50
100
Feature
AIFM3
LZTR1
PI4KA
CRKL
SNAP29
SERPIND1
Cell type (CURIE ontology)
Hepatocyte
Intrahepatic cholangiocyte
Intestinal crypt stem cell of large intestine
Enterocyte
Enterocyte of epithelium of large intestine
Goblet cell
Large intestine goblet cell
Intestinal crypt stem cell
Transit amplifying cell of large intestine
Epithelial cell
Basal cell
Epithelial cell of uterus
Conjunctival epithelial cell
Transit amplifying cell of small intestine
Ciliated cell
Bladder urothelial cell
Respiratory goblet cell
Keratinocyte
Duct epithelial cell
Paneth cell of epithelium of small intestine
Secretory cell
Basal cell of prostate epithelium
Luminal epithelial cell of mammary gland
Club cell
Ionocyte
Kidney epithelial cell
Keratocyte
Lung ciliated cell
Pancreatic ductal cell
Ciliated epithelial cell
Intestinal enteroendocrine cell
Stem cell
Intestinal crypt stem cell of small intestine
Type II pneumocyte
Type I pneumocyte
Corneal epithelial cell
Medullary thymic epithelial cell
Salivary gland cell
Epithelial cell of lacrimal sac
Acinar cell of salivary gland
Duodenum glandular cell
Pancreatic acinar cell
Paneth cell of epithelium of large intestine
Serous cell of epithelium of trachea
Enterocyte of epithelium of small intestine
Type B pancreatic cell
Pancreatic PP cell
Pancreatic A cell
Myoepithelial cell
Serous cell of epithelium of bronchus
Tracheal goblet cell
Intestinal tuft cell
Eye photoreceptor cell
Luminal cell of prostate epithelium
Mucus secreting cell
Small intestine goblet cell
Mesothelial cell
Pigmented ciliary epithelial cell
Pancreatic D cell
Retinal pigment epithelial cell
Pulmonary ionocyte
Surface ectodermal cell

Supplementary Figure S5. Application of cell-type gene expression signatures to spatial indexing data. A. i. Original 8 clusters identified by BayesSpace. ii-iv. Gene expression profiles representing epithelial, stromal, endothelial, fibroblast, and myCAF cells. **B.** Gene expression profiles representative of (i) M1 macrophages, (ii) M2 macrophages, (iii) myeloid DCs, (iv) monocytes, (v) NK cells, and (vi) plasmacytoid DCs. **C.** Gene expression profiles representative of (i) T follicular helper cells, (ii) mature B cells, (iii) CD8 cytotoxic cells, (iv) cytotoxic granules, and (v) CCR7 and ligands CCL19 and CCL21, which are involved in recruitment of mature DCs to lymphoid tissue. **D.** Correlation of the average JAV-Immuno signature vs TLS signature across 4587 Visium spots indicated that the JAV-immuno–expressing spots also contain TLS. Best-fit line (blue) shows a positive correlation (R^2=0.72; p<5.29E−244]).

DC, dendritic cell; **myCAF**, myofibroblastic-like cancer-associated fibroblast; **NK**, natural killer; **TLS**, tertiary lymphoid structures.

A

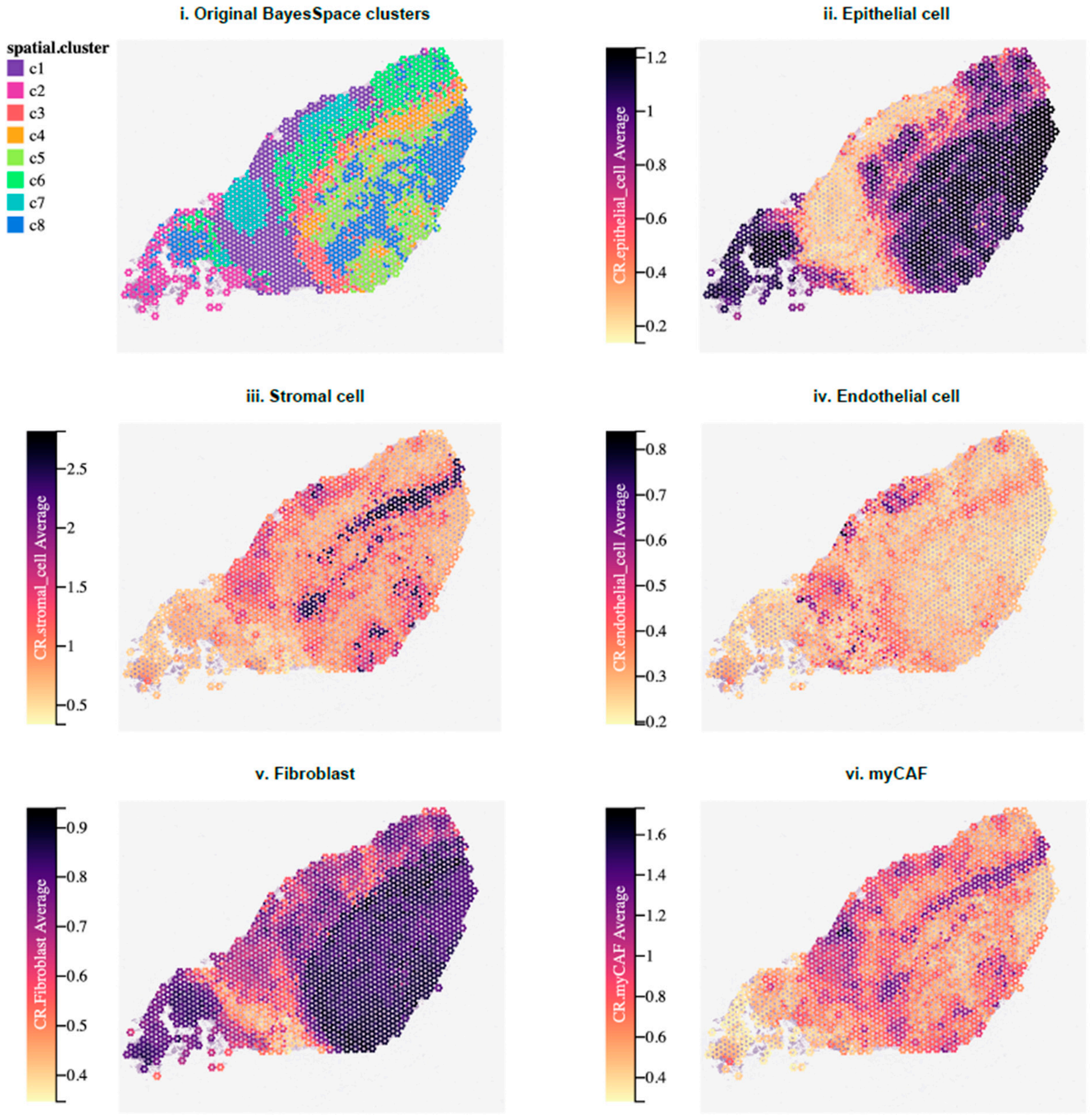

B

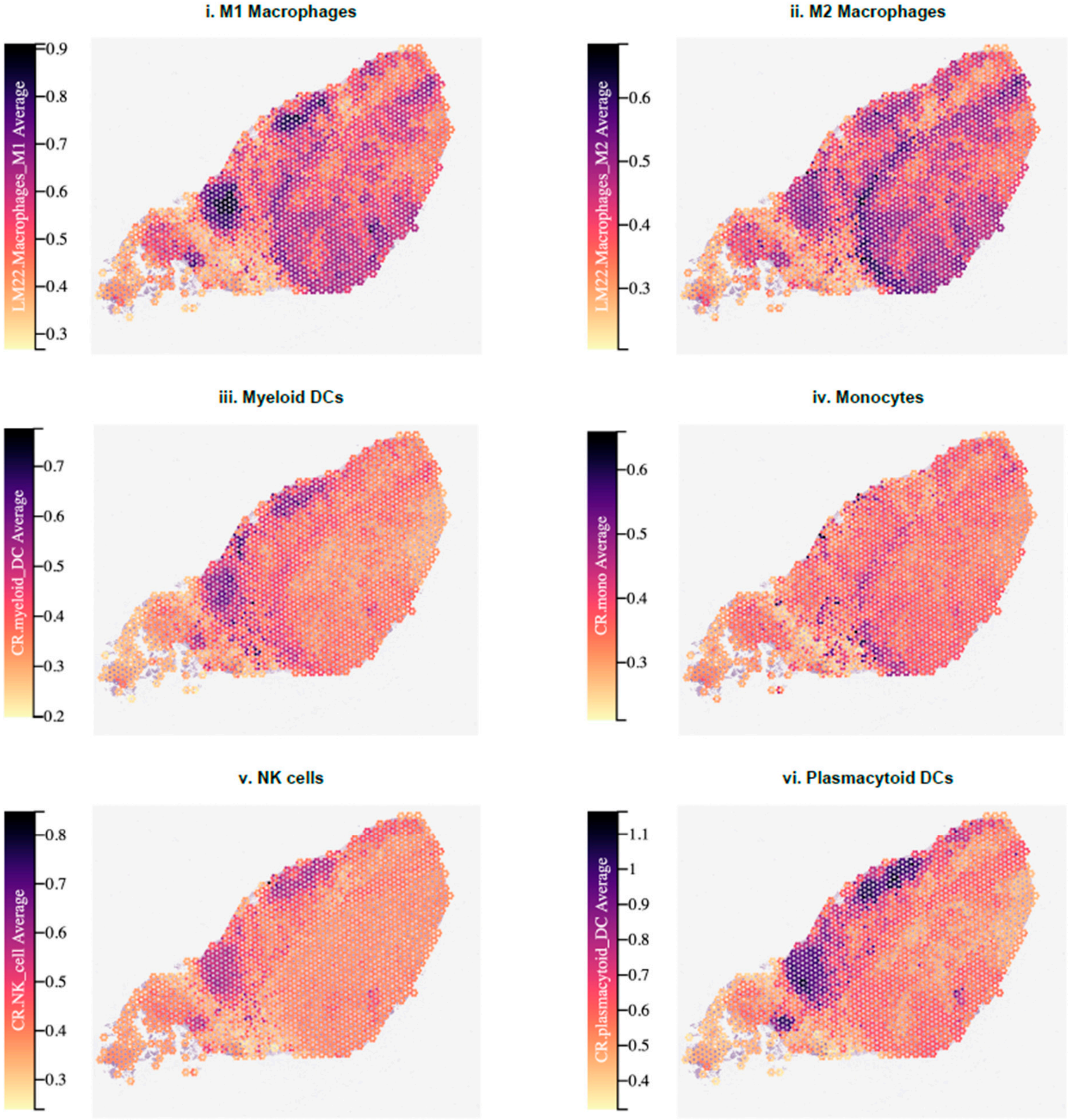
i. M1 Macrophages
LM22.Macrophages_M1 Average
ii. M2 Macrophages
LM22.Macrophages_M2 Average
iii. Myeloid DCs
CR.myeloid_DC Average
iv. Monocytes
CR.mono Average
v. NK cells
CR.NK_cell Average
vi. Plasmacytoid DCs
CR.plasmacytoid_DC Average

C

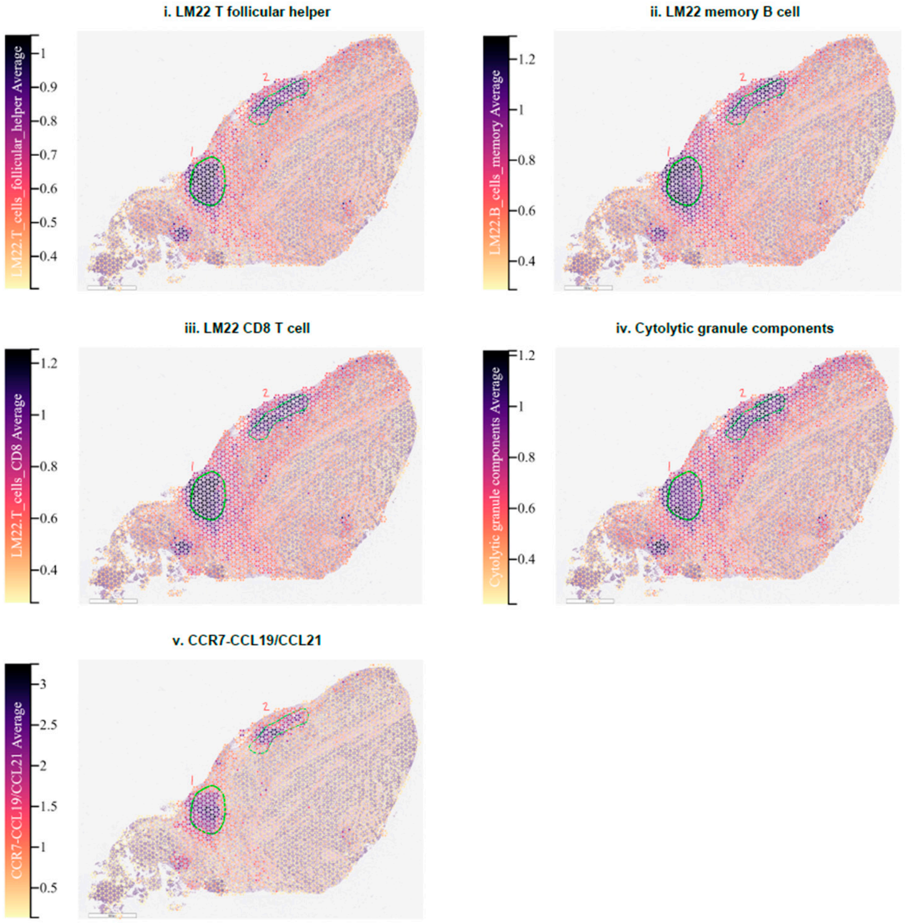

i. LM22 T follicular helper
LM22 T_cells_follicular_helper Average
ii. LM22 memory B cell
LM22 B_cells_memory Average
iii. LM22 CD8 T cell
LM22 T_cells_CD8 Average
iv. Cytolytic granule components
Cytolytic granule components Average
v. CCR7-CCL19/CCL21
CCR7-CCL19/CCL21 Average

D

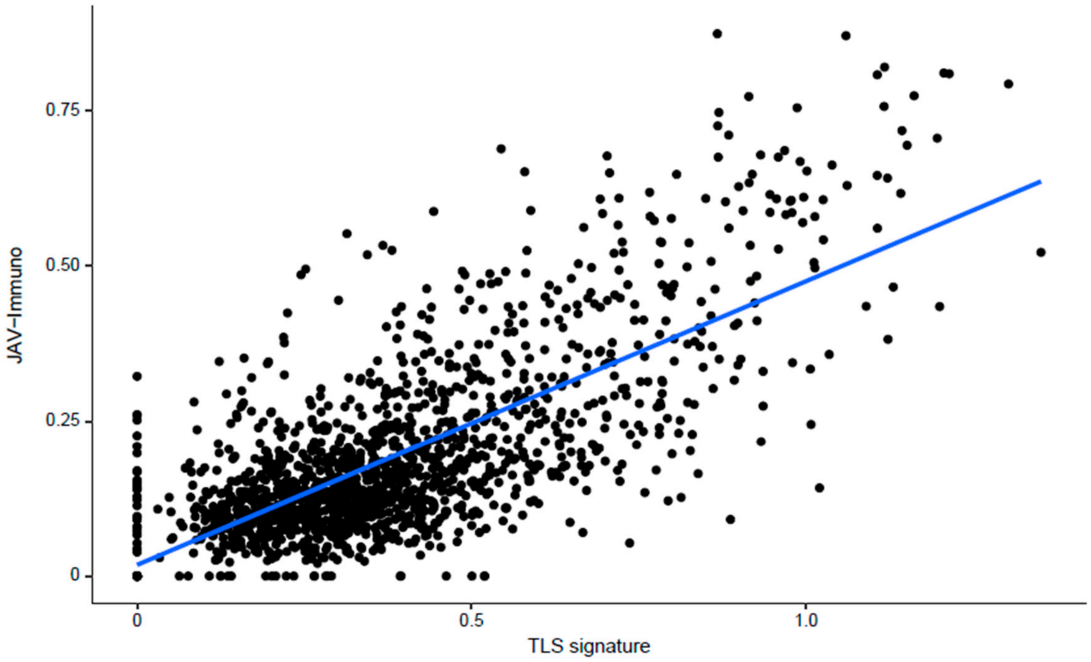

JAV−Immuno
0.75
0.50
0.25
0
0
0.5
1.0
TLS signature

Supplementary Figure S6. Distribution of gene expression level. Genes in the selected marker set were pre-filtered to separate out genes that were expressed in ≤50% of JAVELIN Bladder 100 tumor samples and/or had a coefficient of variation of ≤5%. **TPM**, transcript count per million.

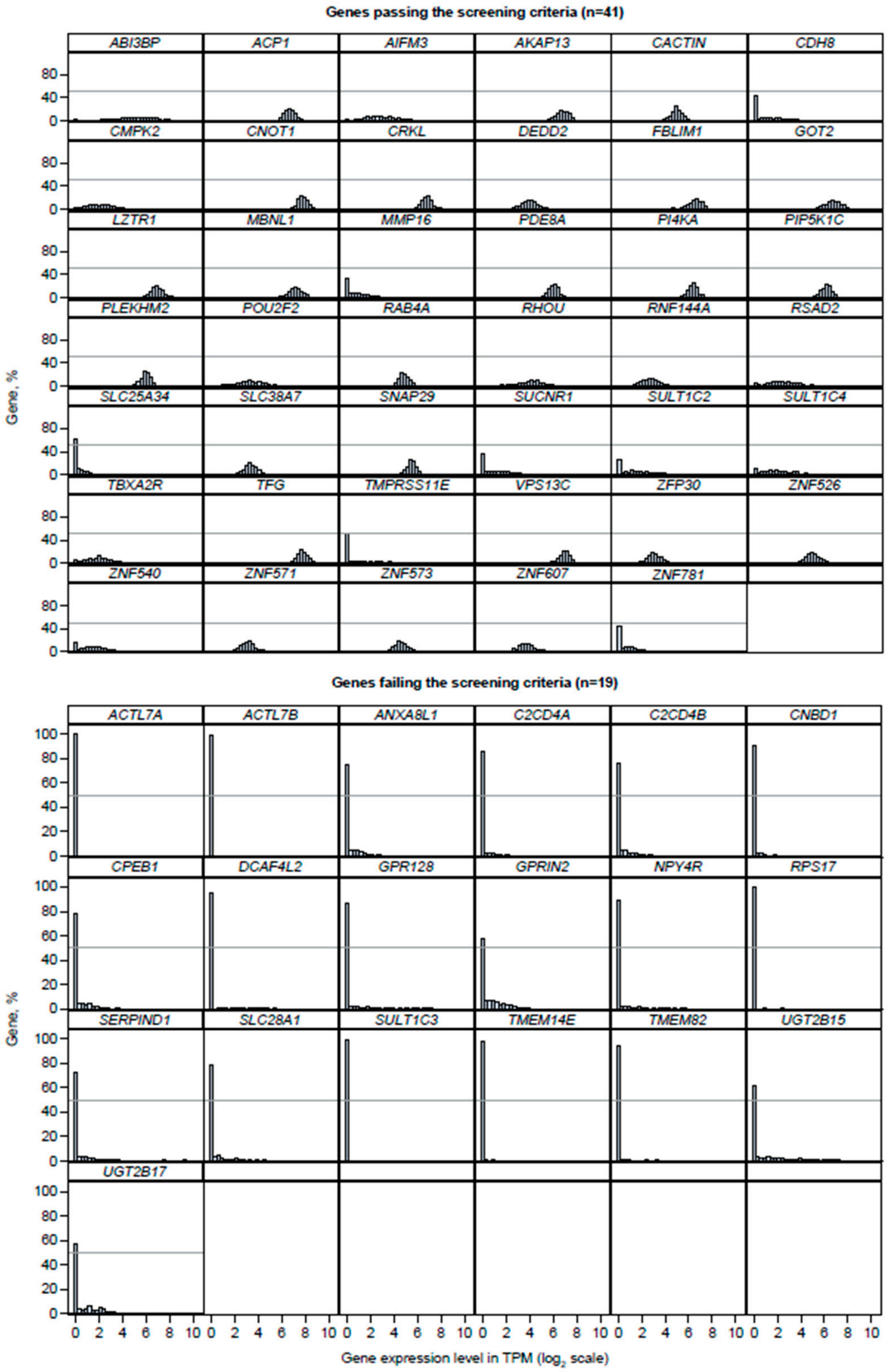

Supplement: Supplementary file 1 [file cancers-17-02332-s001.zip › cancers-3609635-supplementary.pdf]
